# Twelve Months Down the Line: do we know Anything more about the Presence of the SARS-CoV-2 in Human Semen?

**DOI:** 10.1055/s-0041-1729948

**Published:** 2021-05-12

**Authors:** Walter Cardona Maya, Stefan du Plessis

**Affiliations:** 1 Reproduction Group, Department of Microbiology and Parasitology, Medical School, University of Antioquia, Antioquia, Colombia; 2 Department of Basic Sciences, College of Medicine, Mohammed Bin Rashid University of Medicine and Health Sciences, Dubai, United Arab Emirates

Dear Editor,

During the last year, the SARS-CoV-2 infectious agent of COVID-19 has caused more than 75 million infections and 1.5 million deaths worldwide. It has been observed to affect the respiratory tract and exert multiple effects on the human body, including the male reproductive tract. To date, it is understood that although the general infection rate is similar in men and women (50%), deaths in the male population are higher. 1 It has even been reported that the prostate expresses the virus receptor, which could negatively affect the seminal quality and could have a severe effect on human reproduction. 2 3 Therefore, this commentarýs objective was to analyze the scientific data published to date on the presence of the virus in semen.

**Fig. 1:**
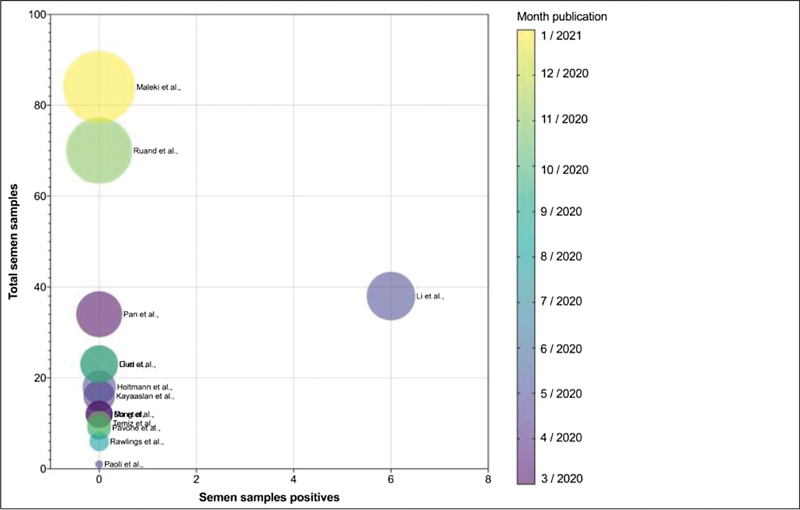
SARS-CoV-2 in semen samples. Total semen samples (the size of each bubble is proportional), positive samples, and date publication (color).

A literature search of the PubMed and Google Scholar databases was performed using the following keywords: COVID-19, SARS-CoV-2, seminal, semen, and sperm. To date, only fourteen articles (both originals and case report) 4 5 6 7 8 9 10 11 12 13 14 15 16 17 described on the presence of the virus in semen samples. Of these, only a single study described to have identified the virus to be present in six semen samples from SARS-CoV-2 positive patients. 4 None of the remaining 13 studies detected the virus in the semen of 351 COVID-19 positive men ( Fig. 1 ).

Based on current evidence, although the virus could be causing adverse effects in the male reproductive tract, its presence in semen continues to be debated because current evidence suggests that the probability of being in semen is very low (1.68%). Consequently, the likelihood of infection through sexual intercourse would be extremely weak. Therefore, as a matter of urgency, further studies on this matter should be pursued.
